# Referential Choice: Predictability and Its Limits

**DOI:** 10.3389/fpsyg.2016.01429

**Published:** 2016-09-23

**Authors:** Andrej A. Kibrik, Mariya V. Khudyakova, Grigory B. Dobrov, Anastasia Linnik, Dmitrij A. Zalmanov

**Affiliations:** ^1^Department of Typology and Areal Linguistics, Institute of Linguistics, Russian Academy of SciencesMoscow, Russia; ^2^Department of Theoretical and Applied Linguistics, Lomonosov Moscow State UniversityMoscow, Russia; ^3^Neurolinguistics Laboratory, National Research University Higher School of EconomicsMoscow, Russia; ^4^Consultant PlusMoscow, Russia; ^5^Linguistics Department, University of PotsdamPotsdam, Germany

**Keywords:** referential choice, non-categoricity, machine learning, cross-methodological approach, discourse production

## Abstract

We report a study of referential choice in discourse production, understood as the choice between various types of referential devices, such as pronouns and full noun phrases. Our goal is to predict referential choice, and to explore to what extent such prediction is possible. Our approach to referential choice includes a cognitively informed theoretical component, corpus analysis, machine learning methods and experimentation with human participants. Machine learning algorithms make use of 25 factors, including referent’s properties (such as animacy and protagonism), the distance between a referential expression and its antecedent, the antecedent’s syntactic role, and so on. Having found the predictions of our algorithm to coincide with the original almost 90% of the time, we hypothesized that fully accurate prediction is not possible because, in many situations, more than one referential option is available. This hypothesis was supported by an experimental study, in which participants answered questions about either the original text in the corpus, or about a text modified in accordance with the algorithm’s prediction. Proportions of correct answers to these questions, as well as participants’ rating of the questions’ difficulty, suggested that divergences between the algorithm’s prediction and the original referential device in the corpus occur overwhelmingly in situations where the referential choice is not categorical.

## Introduction

As we speak or write, we constantly mention various entities, or referents. The process of mentioning referents is conventionally called *reference*. When the speaker’s/writer’s decision to mention a referent is in place, another discourse phenomenon becomes relevant: *referential choice* that is the process of choosing an appropriate linguistic expression for the referent in question. The question of reference per se, that is of how and why a speaker/writer decides which referent to mention at a given place in discourse, is out of the scope of this paper (cf. the point of Gatt et al., 2014, p. 903, that referential choice is not directly related to the likelihood with which a referent is mentioned), that referential choice is not directly related to the likelihood with which a referent is mentioned). The focus of this study is the phenomenon of referential choice: we explore what guides a speaker/writer in choosing a linguistic expression when s/he has already made a decision to mention a certain referent.

The approach to referential choice adopted in the present study relies on earlier work by Chafe (1976, 1994), Givón (1983), Fox (1987), Tomlin (1987), Ariel (1990), and Gundel et al. (1993). These and other theoretical approaches assumed some kind of a cognitive characterization of a referent that underlies referential choice, such as givenness, topicality, focusing, accessibility, salience, prominence, etc. In terms of the cognitive model developed by Kibrik (1996, 1999, 2011) referential choice is governed by *activation in working memory*. In that model reference per se is claimed to be associated with a distinct cognitive phenomenon of *attention*. Attention and working memory are two related but distinct neurocognitive processes (Cowan, 1995; Awh and Jonides, 2001; Engle and Kane, 2004; Awh et al., 2006; Repovš and Bresjanac, 2006; Shipstead et al., 2015). Accordingly, reference and referential choice, as linguistic manifestations of attention and activation, are related but distinct processes (see Kibrik, 2011, Chap. 10).

As is widely held since Chafe (1976) and Givón (1983), the more given (or salient, accessible) a referent is to the speaker at the moment of reference, the less coding material it requires. In terms of the cognitive model we assume, the main law of referential choice can be formulated as follows:

• If the referent’s activation in the speaker’s working memory is high, use a reduced referential device. If the referent’s activation in the speaker’s working memory is low, use a lexically full referential device.

Thus the *basic*, coarse-grained referential choice is between reduced (or attenuated) and lexically full referential devices. In the case of English, it is the distinction between pronouns (personal and possessive), on the one hand, and a variety of full noun phrases, on the other. This distinction is the first level of granularity in the domain of referential options, and all scales and hierarchies that relate givenness (or equivalent concepts) to referential forms (Givón, 1983; Ariel, 1990; Gundel et al., 1993) acknowledge this basic distinction, even though they involve a greater detail in the taxonomy of referential devices. The second level distinction in the domain of referential options is between proper names and descriptions (Anderson and Hastie, 1974; Ariel, 1990; McCoy and Strube, 1999; Poesio, 2000; Heller et al., 2012). There are also further levels of distinction related to varieties of proper names and especially descriptions. In the present study, we mostly concentrate on the first level distinction between pronouns and full noun phrases, and will look briefly into the second level distinction between proper names and descriptions. Our focus is thus different from most work in the current tradition or referring expression generation (REG or GRE, beginning from Dale, 1992 and reviewed in Krahmer and van Deemter, 2012), primarily addressing various types of descriptions. Interestingly, however, Reiter and Dale (2000) recognize that the choice of the “form of referring expressions” (that is, the choice between pronouns, proper names, and descriptions) is the primary one. Krahmer and van Deemter (2012, p. 204) also suggest that first “the form of a reference is predicted, after which the content and realization are determined”.

This study is based on a corpus of written English, specifically newspaper (Wall Street Journal) texts. The corpus is annotated in accordance with the MoRA (Moscow Reference Annotation) scheme, detailed in Section “Materials and Methods” below. We assume that written media texts are a good testing ground for our approach. Specific aspects of referential processes differ across various discourse modes and types (see e.g., Fox, 1987; Toole, 1996; Strube and Wolters, 2000; Efimova, 2006; Garrod, 2011), but the basic cognitive principles of referential choice must be shared by all users of a given language and apply to various discourse types.

Example (1) (from the WSJ corpus we explore) illustrates the major referential options.

(1) But beyond this decorative nod to tradition, Ms. Bogart and $\underset{¯}{\underset{¯}{\text{company}}}$ head off in a stylistic direction that all but transforms Gorky’s naturalistic drama into something akin to, well, farce. The director’s attempt to Ø force some Brechtian distance between her $\underset{¯}{\underset{¯}{\text{actors}}}$ and $\underset{¯}{\underset{¯}{\text{their}}}$ characters frequently backfires with performances that are unduly mannered. Not only do $\underset{¯}{\underset{¯}{\text{the actors}}}$ stand outside $\underset{¯}{\underset{¯}{\text{their}}}$ characters and $\underset{¯}{\underset{¯}{\text{Ø}}}$ make it clear $\underset{¯}{\underset{¯}{\text{they}}}$ are at odds with them, but $\underset{¯}{\underset{¯}{\text{they}}}$ often literally stand on $\underset{¯}{\underset{¯}{\text{their}}}$ heads.

Two referents recur a number of times in (1). They are emphasized with two different kinds of underlining: Ms. Bogart and $\underset{¯}{\underset{¯}{\text{the actors}}}$. The first referent is mentioned with a proper name (title plus last name), a description (the director), as well as with a pronoun (her) and a zero (in an infinitival construction). The second referent is mentioned by two different descriptions ($\underset{¯}{\underset{¯}{\text{company}}}$ and $\underset{¯}{\underset{¯}{\text{actors}}}$), pronouns ($\underset{¯}{\underset{¯}{\text{they}}}$, $\underset{¯}{\underset{¯}{\text{their}}}$), and a zero (in a coordinate construction). (In written English, zeroes are not a part of discourse-based referential choice, but they can serve as antecedents; see discussion in Section “Materials and Methods”.)

What factors influence actual referential choices in discourse? In usual face-to-face conversation, an entity sometimes become visually available to the interlocutors (via shared attention), and that may be enough for using an exophoric pronoun without any antecedent (see e.g., Cornish, 1999). In written discourse, however, factors affecting referential choice are mostly associated with (i) the referent’s internal properties and (ii) the discourse context. Referent’s internal properties vary from most inherent, such as animacy, to more fluid, such as being or not being the protagonist of the current discourse. The factors of discourse context are diverse and include the following groups:

• those related to a prospective anaphor, such as the ordinal number of the given mention in the given discourse
• those related to the antecedent’s properties, such as its grammatical role (subject, object, etc.)
• those related to discourse structure, such as the distance between the anaphor and the antecedent, measured in the number of clauses or paragraphs.

Referential choice thus belongs to a large family of *multi-factorial processes*, generally characteristic of language production. Most of the factors employed in our study, such as animacy, grammatical role, or distance to antecedent, have been proposed in prior literature, in particular (Paducheva, 1965; Chafe, 1976; Grimes, 1978; Hinds, 1978; Clancy, 1980; Marslen-Wilson et al., 1982; Givón, 1983; Brennan et al., 1987; Fox, 1987; Tomlin, 1987; Ariel, 1990; Gernsbacher, 1990; Gordon et al., 1993; Dahl and Fraurud, 1996; Kameyama, 1999; Yamamoto, 1999; Strube and Wolters, 2000; Arnold, 2001; Stirling, 2001; Tetreault, 2001; Arnold and Griffin, 2007; Kaiser, 2008; Fukumura and van Gompel, 2011, 2015; Fukumura et al., 2013; Fedorova, 2014; Rohde and Kehler, 2014, i.a.). There is no room here to review this literature in detail, but many of these studies are discussed in Kibrik (2011); see also recent reviews in van Deemter et al. (2012) and Gatt et al. (2014). In some of the above-mentioned studies one of the factors was emphasized, while others were ignored or shaded. We find it important to take as many relevant factors as possible into account, as they actually operate in conjunction.

**FIGURE 1:**
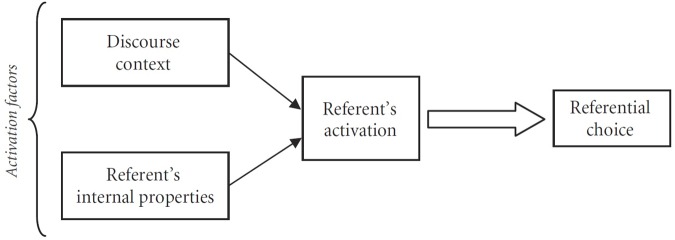
**Cognitive model of referential choice (cf. Kibrik, 2011, p. 61)**.

Within the cognitive model we assume, these factors are interpreted as *activation factors*, contributing to the cumulative current referent’s activation. This cognitive model of referential choice is depicted in **Figure 1** (see further specification of the model in Sections “Discussion: Referential Choice Is Not Always Categorical” and “Experimental Studies of Referential Variation”). Two kinds of activation factors operate in conjunction and determine a referent’s current degree of activation, which in turn predicts referential choice.

In Kibrik (1996, 1999) a simple mathematical model was developed, capturing the multiplicity of factors and their relative contributions to referent activation and, therefore, to the ensuing referential choice. In those studies referent’s current activation level was assessed numerically, as a so-called activation score ranging from a minimal to a maximal value. In this paper, in contrast, we present a study based on machine learning techniques, in which we supply activation factors’ values to algorithms and obtain predictions of referential choice as an output. Therefore, the activation component remains hidden within the algorithm, and only mappings of activation factors upon referential options are explicit. In this respect this study is similar to most other studies or referential choice cited above, as well as to the studies based on annotated referential corpora, such as Poesio and Artstein (2008) and Belz et al. (2010). Still we find it important to keep the larger picture in mind and recognize that in the human cognitive system referent’s activation level mediates between the relevant factors and the actual referential choice.

We pursue two goals in this paper. The first goal is *to predict referential choice* as reliably as possible. We explore a corpus of English written discourse and use machine learning techniques to predict referential choice maximally close to the original texts. This part of the study is reported in Section “Corpus-Based Modeling”. In the course of this work it is found that even well-trained algorithms sometimes diverge from the original referential choices in the corpus texts.

That brings us to the second goal of our research: is 100% accurate prediction of referential choice possible in principle? In addressing this question, we consider the possibility that certain instances of divergence between the predicted and original forms may be due to the *incomplete categoricity* of referential choice. In Section “Experimental Studies of Referential Variation”, we submit the instances of divergence to an experimental assessment by human participants, in order to see whether people accept referential variation in the spots where divergences take place.

The discussion of our findings and concluding remarks follow in Section “General Discussion”.

## Corpus-Based Modeling

### Related Work

During the last twenty years or so a number of corpus resources for studies of coreference and reference production has appeared, including MUC-6/-7 (Chinchor and Sundheim, 1995; Grishman and Sundheim, 1995; Chinchor and Robinson, 1997), the ASGRE challenge (Gatt and Belz, 2008), the GNOME corpus (Poesio, 2000, 2004), the ARRAU corpus (Poesio and Artstein, 2008), and the GREC-08, -09, -10 challenges (Belz and Kow, 2010; Belz et al., 2008, 2009). Among these, the series of studies conducted for the GREC (Generating Referring Expressions in Context) challenges were somewhat similar in their goals to the present study: they predicted the form of a referring expression (common noun, name/description, pronoun, or “empty” reference) in Wikipedia articles about cities, countries, rivers, and people. One of the successful algorithms, a memory-based learner (Krahmer et al., 2008), was able to predict the correct type of referring expression in 76.5% of the cases. Krahmer et al. (2008) used automatic language processing tools to mark the following parameters for every entity: competition, position in the text, syntactic and semantic category, local context (POS tags), distance to the previous mention in sentences and NPs, main verb of the sentence, and syntactic patterns of three previous mentions. The systems in the 2010 GREC challenge used various sets of factors and machine learning techniques; for example, Greenbacker and McCoy (2009) used such features as competition, parallelism, and recency. The best system’s precision in the prediction task reached 82-84%. Zarrieß and Kuhn (2013) report a similarly high prediction accuracy in their study inspired by the GREC tasks on a corpus of German robbery reports. Crucial differences of the present work from the GREC studies are that, first, all referents are considered, not just the main topic referent of each article, and, second, semantic discourse structure is taken into account. Recent reviews providing detailed accounts of corpus-based studies of reference production can be found in Krahmer and van Deemter (2012) and Gatt et al. (2014).

Early modeling studies by Kibrik (1996, 1999) were mentioned in Section “Introduction”. Grüning and Kibrik (2005) applied the neural networks method of machine learning to the same small dataset as in Kibrik (1999); that study showed that machine learning is in principle appropriate for modeling multi-factorial referential choice and raised the question of creating a much larger and statistically valid corpus designed for referential studies. Several studies of our group addressed a corpus of Wall Street Journal texts, somewhat larger than the one used in the present paper (Kibrik and Krasavina, 2005; Krasavina, 2006) and used the annotation scheme proposed in Krasavina and Chiarcos (2007). More recently we developed the MoRA (Moscow Reference Annotation) scheme and conducted machine learning studies on the corpus data, looking into the basic referential choice (two-way choice between pronouns and full NPs) and the three-way choice between pronouns, proper names, and descriptions (Kibrik et al., 2010; Loukachevitch et al., 2011). Compared to our previous publications, in the present study we have substantially improved the quality of corpus annotation and modified the annotation scheme and the machine learning methods.

A number of studies emphasized the role of discourse structure in referential choice. In his classical work, Givón (1983) introduced the concept of linear distance from an anaphor back to the antecedent, measured in discourse units such as clauses. Other studies (Hobbs, 1985; Fox, 1987; Kibrik, 1996; Kehler, 2002) underlined the contribution of the semantic structure of discourse, including the hierarchical structure. Several models of discourse-semantic relations have been proposed in the recent decades (see Hobbs, 1985; Polanyi, 1985; Wolf et al., 2003; Miltsakaki et al., 2004; Joshi et al., 2006, i.a.), one of the best known being Rhetorical Structure Theory (RST) (Mann and Thompson, 1987; Taboada and Mann, 2006). RST represents text as a hierarchical structure, in which each node corresponds to an elementary discourse unit (EDU), roughly equaling a clause. Fox (1987) demonstrated a possible connection between reference and RST-based analysis of dicourse, and Kibrik (1996) introduced the measurement of rhetorical distance (RhD) that captures the length of path between an anaphor EDU and the antecedent EDU along the rhetorical graph; see Section “Materials and Methods”. In a neural networks-based study (Grüning and Kibrik, 2005) it was also found that RhD was an important factor. Experimental studies of Fedorova et al. (2010b, 2012) demonstrated that RhD is a relevant factor affecting referent activation in working memory, as well as reference resolution in the course of discourse comprehension.

The WSJ MoRA 2015 corpus employed in this paper (we used the name “RefRhet corpus” for earlier versions in previous publications) is based on a subset of texts of the RST Discourse Treebank, developed by Daniel Marcu and his collaborators (Carlson et al., 2002). This allows us to combine our own annotation (see Materials and Methodsith the rhetorical annotation produced by the Marcu’s team, and to compute RhD on the basis of their annotation. To the best of our knowledge, corpora intended for referential studies and containing discourse semantic structure annotation are few on the market; cf. the German corpus Stede and Neumann (2014). An English language resource comparable to ours in using discourse semantic structure as a part of referential annotation is the so-called C-3 corpus outlined in Nicolae et al. (2010). As these authors correctly state,

“the most widely known coreference corpora < … > are annotated with relations between entities, not between discourse segments. The most widely known coherence corpora are Discourse GraphBank (Wolf & al., 2003), RST Treebank (Carlson & al., 2002), and Penn Discourse Treebank (Prasad & al., 2008), none of which was annotated with coreference information.” (Nicolae et al., 2010, p. 136).

Nicolae et al.’s (2010) project is similar to ours in that they picked an already existing corpus annotated for discourse semantic relations and added further annotation for the purposes of modeling reference. Unlike us, however, they chose not the RST Discourse Treebank but the Discourse GraphBank of Wolf et al. (2003). The latter corpus is based on a less constrained kind of discourse representation compared to RST; see discussion in Marcu (2003), Wolf et al. (2003), and Wolf and Gibson (2003).

Referential annotation added by Nicolae et al. (2010) includes primarily types of entities (persons, organizations, locations, etc.), referential status (specific, generic, etc.) and referential form (pronoun, proper name, description, etc.). The number of entity types is greater than in our annotation scheme, but in general there are much fewer parameters involved. In particular, it seems that the syntactic role of anaphors and antecedents is not annotated. Generally Nicolae et al. (2010) followed the ACE (Automatic Content Extraction, 2004) guidelines principles of coreference annotation. They developed their own annotation tool. We are not aware of specific modeling studies based on the C-3 corpus.

A variety of algorithms have been used in computational studies of referential choice. One of the well-known early algorithms is the so-called incremental algorithm that was used by Dale and Reiter (1995) to predict the choice of attributes in descriptions. Modifications of this algorithm include the ones developed by van Deemter (2002) and Siddharthan and Copestake (2004), i.a.. In the 2000s, with the development of corpora for referential studies, researchers began to use classical machine learning algorithms and methodology to analyze some features of referential expressions. For example, in Cheng et al. (2001) the classification task was to determine the NP type, and the corpus annotation was used to train a classifier. The authors used the CART (Classification and Regression Trees) classifier and achieved 67 and 75% accuracy on different text sets by cross-validation procedure. Early corpus- and machine learning-based studies similar to ours in design are Poesio et al. (1999) and Poesio (2000). In the studies related to the GREC challenges (Belz and Varges, 2007; Belz and Kow, 2010), the algorithms had to identify the correct referring expression from a provided set. Participants used various methods and features to perform the task. For example, in 2008 they were: Conditional Random Fields with a set of features encoding the attributes given in the corpus, information about intervening references to other entities, etc. (UMUS system); a set of decision tree classifiers that checked the length of referring expressions and correctness of pronouns (UDEL system); XRCE system that used a great number of features with levels of activation. Other studies applying machine learning specifically to discourse reference include Jordan and Walker (2005), Viethen et al. (2011), and Ferreira et al. (2016). Also, there is a number of studies in which machine learning was used in other language generation tasks, such as prediction of adjective ordering (Malouf, 2000), content selection (Kelly et al., 2009), accent placement (Hirschberg, 1993), sentence planning (Walker et al., 2002), automated generation of multi-sentence texts (Hovy, 1993), as well as other tasks (e.g., Dethlefs and Cuayáhuitl, 2011; Dethlefs, 2014; Stent and Bangalore, 2014).

### Materials and Methods

#### The Corpus

The WSJ MoRA 2015 corpus explored in this study consists of Wall Street Journal articles from the late 1980s, including broadcast news, analytical reviews, cultural reviews, and some other genres. Text length varies from 70 words to about 2000 words, the average length being 375 words. A general quantitative characterization of the WSJ MoRA 2015 corpus appears in **Table 1.**

**Table 1:** The WSJ MoRA 2015 corpus: a quantitative characterization.

Feature	Comment	Number in corpus
Texts		64
Paragraphs		511
Sentences		976
Elementary discourse units (EDU)	EDU segmentation of texts is automatically extracted from the RST Discourse Treebank	2928
Words		23952


Referential annotation of the corpus consists of two parts: annotation of referential devices and annotation of candidate activation factors. We consider these two kinds of annotation in turn.

##### Annotation of referential devices

Referential devices are technically named markables that is those referential expressions that can potentially corefer. Coreferential expressions form a *referential chain*. Non-first members of a referential chain are termed *anaphors* below. The breakdown of markables by type is shown in **Table 2.**

**Table 2:** Types and numbers of markables (referential expressions).

	Type of markable	Comment	Number in corpus
1.	**Reduced referential devices**	Sum of #2 to #7	**1373**
2.	Personal pronouns		495
3.	Possessive pronouns		264
4.	Zeroes		375
5.	Demonstratives		67
6.	Relative pronouns		135
7.	Other		37
8.	**Full noun phrases**	Sum of #9 and #18 minus #27^∗)^	**5042**
9.	**Descriptions**	Sum of #10 to #15	**3517**
10.	The-descriptions		1241
11.	A-descriptions		420
12.	Bare descriptions		1200
13.	Demonstrative descriptions	E.g. *this house*	88
14.	Possessive descriptions	E.g. *his house, the company’s shares*	490
15.	Other		78
	*Special subtypes*		
16.	Attributive descriptions	E.g. *the American president; the first American president who was elected…*	1458
17.	Numeral descriptions	E.g. *the two books*	136
18.	**Proper names**	Sum of #19 to #25^∗)^	**1681**
19.	First names		21
20.	Last names		229
21.	First plus last names		193
22.	Initials plus last names	E.g. *G.W.Bush*	1
23.	Non-persons	Names of countries, organizations, units, etc.	915
24.	Acronyms	E.g. *GE, the US*	277
25.	Other		45
	*Special subtype*		
26.	Titled proper names	E.g. *Mr. Bush*	162
**27.**	**Mix: description plus proper name**	E.g. *President Bush*	**156**
	**TOTAL**		**6415**


*^∗^Special subtypes in lines 16–17 and 26 cross-cut the mutually exclusive subtypes appearing in lines 10–15 and 19–25, respectively, and therefore are not summed with those in the counts shown in lines 9 and 18.*

Note that not every markable in the corpus is actually used for analysis. First, there are 2580 singleton markables that are not linked to any other markable by a coreference relation and are not pertinent to referential choice. (They are nevertheless annotated, as they are taken into account when the values for the factor “distance in markables” are calculated.) In the modeling task we only use those markables that form referential chains. Second, certain types of referential expressions are only considered as antecedents, but not as anaphors in our analysis of referential choice. This concerns the following categories:

‒ indefinite descriptions (introduced by indefinite determiners, such as *a(n), some, few*, etc.);
‒ bare descriptions;
‒ all types of pronouns other than personal and possessive;
‒ first and second person pronouns;
‒ zero references.

In particular, quite common zero references in English only appear in fixed syntactic contexts, such as coordinate, gerundial, and infinitival constructions; at least this applies to the kind of written English we explore (cf. Scott, 2013). Syntactically induced zeroes should not be treated as a discourse-based referential option on a par with third person pronouns and full NPs. At the same time, zeroes make bona fide antecedents, so they must be annotated as markables in a referential corpus^1^. Similar reasoning applies to relative pronouns. In written discourse, nominal demonstratives such as *that* typically refer to situations rather than entities.

In the corpus, there are 777 referential chains that comprise at least one anaphor, meeting the above-listed requirements (i.e., is not a bare description, a zero, etc.). Such chains include 3199 markables used in the modeling tasks. Average chain length is 4.1 markables, and the maximum length of a chain is 52 markables.

We thus address the basic referential choice between third person personal/possessive pronouns and full noun phrases. **Table 3** shows the numbers of anaphors in the corpus.

**Table 3:** Anaphor types.

Anaphor type	Number used for analysis
Third person pronouns (personal or possessive)	585 (26.0%)
Descriptions	856 (38.1%)
Proper names	807 (35.9%)
Total	2248 (100%)


##### Annotation of candidate activation factors

The second part or referential annotation addresses candidate activation factors that is parameters that are potentially useful for the prediction of referential choice. The complete list of candidate factors used in this study is shown in **Table 4.** For each factor, its values included in the study are listed after a colon. Most of the factors’ values are derived from the MoRA scheme annotation, but some are computed automatically.

**Table 4:** Candidate factors of referential choice.

**(1) Referent’s factors**
• Animacy: animate, inanimate, collective (*for such entities as organizations*) • Gender (for animate referents only): masculine, feminine, mixed (*for groups of people with various or unspecified gender*) • Person: 1, 2, 3 • Number: singular, plural • Protagonism: *numeric value*
**(2) Anaphor’s factors**
• Ordinal number of referent mention in the referential chain: *integer* • Type of phrase: noun phrase, prepositional phrase • Grammatical role: subject, direct object, indirect object, oblique (with preposition), attribute, *’s*-genitive, *of*-genitive, postpositive specification
**(3) Antecedent’s factors**
• Type of phrase (values same as in the section “Anaphor’s factors”) • Grammatical role (values same as in the section “Anaphor’s factors”) • Referential form: ∘ pronoun: personal, possessive, demonstrative, relative, zero ∘ description: a-description, the-description, bare description, demonstrative description, possessive description ∘ attributive ∘ numeral ∘ proper name: first, last, first and last, initials and last, non-person, acronym ∘ Antecedent length, in words: *integer*
**(4) Distances between anaphor and antecedent**
• Distance in words: *integer* • Distance in all markables: *integer* • Number of markables in chain from the anaphor back to the nearest full NP antecedent: *integer* • Linear distance in EDUs: *integer* • Rhetorical distance (RhD) in elementary discourse units: *integer* • Distance in sentences: *integer* • Distance in paragraphs: *integer*

In **Table 4**, the factors are listed in four groups. In the terms of **Figure 1**, the group 1 factors roughly correspond to the “Referent’s internal properties” activation factors, while group 2–4 factors to the “Discourse context” activation factors. For the sake of brevity, the logic of factors is somewhat simplified in **Table 4.** In particular, most factors include the value “other” that we omit here. Several of the factors call for clarifying comments.

Protagonism means referent’s centrality in discourse. Two models of protagonism were used (Linnik and Dobrov, 2011): in the first one, to each referent corresponds the ratio of its referential chain length to the maximal length of a referential chain in the text; in the second model, to each referent corresponds the ratio of its chain length to the gross number of markables in the text. In both instances, the most frequently mentioned referent is the same, but relative weights of referents may be different.

Regarding the “Type of phrase” factor, it is important to explain why we consider prepositional phrases (such as *of the president* or *with her*) a particular type of phrase, rather than a combination of a preposition with a referential device (noun phrase). First, referential choice may depend on whether the antecedent or the anaphor is a plain noun phrase, or a noun phrase subordinate to a preposition (that is, constitutes a prepositional phrase); so this information must be retained. Second, consider English ’*s-* and *of*-genitives. The former are inflectional word forms and cannot be divided into a referential device and a separate unit, and it is reasonable to treat the two different kinds of genitives in the same way. More generally, in many languages, equivalents of English prepositions would be case endings, and nobody would deduct these from referential expressions.

**FIGURE 2:**
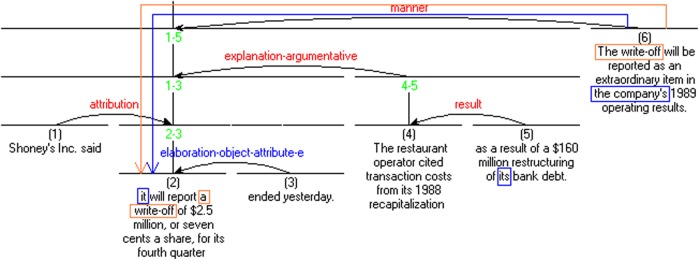
**Example of a rhetorical graph from RST Discourse Treebank with examples of RhD computation.** The referent ‘the write-off’ is mentioned in units #2 and #6. Linear distance from #6 back to #2 equals 4. Rhetorical distance (RhD) from #6 to #2 is just 1, as these two nodes are immediately connected to each other in the RST graph, and one only needs one horizontal step along the graph to reach #2. The anaphor *the company* found in unit #6 has its closest linear antecedent in unit #5. However, its closest rhetorical antecedent is again found in #2, directly connected to the anaphor unit #6. Arrows indicate paths along the RST graph one needs to travel to reach an antecedent.

Most of the distance factors are identifed for the closest linear antecedent. In contrast, RhD is computed from the anaphor back to the nearest rhetorical antecedent along the hierarchical graph. **Figure 2** presents an example of the RST Discourse Treebank annotation, as well as illustrates the difference between the linear and the rhetorical antecedents, and the corresponding distances. Principles of RhD computation were outlined in Kibrik and Krasavina (2005).

In all, 25 potentially relevant activation factors are extractable from the annotated WSJ MoRA 2015 corpus; these are independent variables in the computational models discussed below. The parameter *anaphor’s referential form* is the predicted, or dependent, variable.

Each text of the WSJ MoRA 2015 corpus was annotated by two different annotators, and each pair of annotations was compared with the help of a special script that identified divergences. All problematic points were fixed by an expert annotator. The corpus was subsequently cross-checked with a variety of techniques and corrected by the members of our team.

**FIGURE 3:**
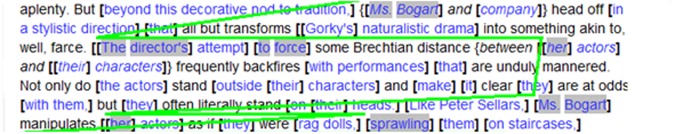
**A sample of text annotation in MMAX2**.

**Figure 3** provides a screenshot from the MMAX2 annotation tool (Müller and Strube, 2006) for the same text excerpt that was used as Example (1) in Section “Introduction”. Here, all expressions that refer to “Ms. Bogart” are highlighted and grouped into one referential chain with lines that mark coreference.

A special property of the MoRA scheme is the annotation of *groups*. A group is a set of markables that, collectively, serve as an antecedent of an anaphor. In **Figure 3**, two groups are present, marked with curly brackets and with italics: {[*Ms. Bogart*] *and* [*company*]} and {*between* [[*her*] *actors*] *and* [[*their*] *characters*]}. Later on in the text, there is indeed the markable [of the ensemble], the antecedent of which is {[*Ms. Bogart*] *and* [*company*]}.

#### Computational Modeling

In this study we use the system Weka^2^ (see Hall et al., 2009) that includes many algorithms of machine learning, as well as automated means of algorithms’ evaluation. Several types of algorithms, or classifiers, are used. We consider the wide variety of used algorithms as an important methodological property of our study, distinguishing it from most other studies in reference production.

First, we use a logical algorithm (decision trees C4.5) as it lends itself to natural interpretation. Second, we use logistic regression because its results often exceed those of logical algorithms in quality. In addition, we use the so-called classifier compositions: bagging (Breiman, 1996) and boosting (Freund and Schapire, 1996). These composition algorithms use, as a source of their parameters, another machine learning algorithm that we will call the base algorithm. Using the base algorithm, composition algorithms construct multiple models and combine their results. As was shown in several experimental studies (for example, Schapire, 2003), composition algorithms or their modifications “performed as well or significantly better than the other methods tested” (Schapire, 2003, p. 162).

In the boosting algorithm the base algorithm undergoes optimization. An adaptation of classifiers is performed, that is, each additional classifier applies to the objects that were not properly classified by the already constructed composition. After each call of the algorithm the distribution of weights is updated. (These are weights corresponding to the importance of the training set objects.) At each iteration the weights of each wrongly classified object increase, so that the new classifier focuses on such objects. Among the boosting algorithms, AdaBoost was used in our modeling with C4.5 as the base algorithm.

Bagging (from “bootstrap aggregating”) algorithms are also algorithms of composition construction. Whereas in boosting each algorithm is trained on one and the same sample with different object weights, bagging randomly selects a subset of the training samples in order to train the base algorithm. So we get a set of algorithms built on different, even though potentially intersecting, training subsamples. A decision on classification is made through a voting procedure in which all the constructed classifiers take part. In the case of bagging the base algorithm was also C4.5.

In order to control the quality of classification, the cross-validation procedure was used:

(1) The training set is divided into ten parts.
(2) A classifier operates on the basis of nine parts.
(3) The constructed decision function is tested on the remaining part.

The procedure is repeated for all possible partitions, and the results are subsequently averaged. The criterion for choosing both an optimal set of features and an algorithm is *accuracy* that is the ratio of properly predicted referential expressions to the overall amount of referential expressions. As was pointed out above, all the independent variables contained in **Table 4** were treated as candidate factors of referential choice and included into our machine learning studies.

### Results

#### Predicting Basic Referential Choice

The results of modeling the basic choice between reduced and full referential devices are given in **Table 5.** The baseline means the frequency of the most frequent referential option, that is, full noun phrase. If an algorithm always predicted the most frequent option, its accuracy would equal that option’s frequency. **Table 5** also includes information on three additional measures assessing the quality of classification: precision, recall, and F1 (or harmonic mean).

**Table 5:** Prediction of the basic referential choice.

Algorithm	Accuracy	Full NP			Pronoun		
			
		Precision	Recall	F1	Precision	Recall	F1
Baseline	74.0%	74.0%	1	85.0%	0	0	0
C4.5 algorithm	88.9%	91.7%	92.0%	91.9%	77.3%	76.7%	77.0%
Logistic regression	88.6%	91.5%	92.6%	92.1%	78.5%	76.0%	77.2%
Bagging	89.4%	91.9%	93.6%	92.7%	81.0%	76.8%	78.9%
Boosting	89.8%	92.2%	93.6%	92.8%	80.9%	77.4%	79.1%


The results yielded by any of the algorithms surpass the baseline substantially. At the same time, with the given set of factors all the algorithms demonstrate very close results; in particular, the accuracy rate is in the vicinity of 89-90%. The boosting algorithm fairs somewhat better than the others, but its difference from the other algorithms is not statistically significant. (We performed the McNemar’s test of statistical significance, in accordance with the method described in Salzberg, 1997.)

The confusion matrix (i.e., information on the amount of divergent predictions done by a classifier) for the boosting algorithm appears in **Table 6.** The model predicts over 93% of full NPs correctly, but is less effective with respect to pronouns: only 77% are predicted correctly. Such difference in performance can be explained by the class imbalance in the task: machine learning algorithms “prefer” to predict the most frequent class (full NP in our case) and thus achieve higher overall accuracy (Longadge et al., 2013). It is hardly possible to avoid class imbalance in a corpus-based study, in which relative frequencies of tokens consitute an inherent part of the data.

**Table 6:** Confusion matrix for the boosting algorithm, basic referential choice.

	Predicted full NP	Predicted third person pronoun	Total
Original full NP	1556 (93.6%)	107 (6.4%)	1663 (100%)
Original pronoun	132 (22.6%)	453 (77.4%)	585 (100%)


#### Interpreting Decision Trees

Among the machine learning algorithms, decision trees may be particularly telling in explicitly specifying the concrete role of certain factors. For our corpus, a decision tree was generated that comprised 110 terminal nodes each corresponding to a specific prediction rule. Consider the following branch from the decision tree: if the anaphor is a prepositional phrase and its antecedent lies within the same sentence, then it is most probable that a full noun phrase will be chosen, not a pronoun. Of 100 instances observed, only 8 display pronominalization. A typical example can be seen in (2).

(2) Israel has launched a new effort to prove the Palestine Liberation Organization continues to Ø practice terrorism, and thus to persuade the U. S. to break off talks with the group.

This finding is quite surprising, given the closeness of the anaphor to the antecedent. The specific explanation of the finding is yet to be determined, but it is clear that the decision tree algorithms provide a source of new cause-effect generalizations about referential choice that would otherwise remain unrevealed.

#### Factors’ Contribution

What is the role of individual factors to the success of prediction? In order to evaluate such role, we have applied the boosting algorithm to different subsets of factors in order to find out the individual contribution of factors or their combinations. The results are provided in **Table 7.**

**Table 7:** The significance of factors in modeling the basic referential choice (boosting with 50 iterations).

Factors	Accuracy(%)
All factors	89.8
— without animacy	89.4
— without protagonism	89.7
— without the anaphor’s grammatical role	88.3
— without the antecedent’s grammatical role	89.2
— without grammatical role	87.7
— without the antecedent’s referential form	89.4
All non-distance factors only	75.5
— plus distance in all markables	82.5
— plus distances in words and paragraphs	87.2
— plus RhD, distance in words, and distance in sentences	88.7
All distance factors only	83.2


We used a number of distance measurements in this study. The data in **Table 7** suggests that this group of factors is essential for successful prediction. As the distance factors are highly correlated, using any of them increases accuracy dramatically. Accuracy increases further if two or three distance factors are included. The non-distance factors have complex impact on accuracy: eliminating them one by one does not impair prediction significantly, but removing all of them results in a significant decrease of accuracy and is therefore inadvisable.

An earlier study of our group (Loukachevitch et al., 2011) specifically looked into the selection of factors and explored the relationships between them. Models based on various subsets of the factors were tested, and it was demonstrated that none of those models surpassed the full set of factors in classification quality. Deduction of each individual factor led to some deterioration of prediction. This makes us believe that the full set of factors used in our studies can hardly be reduced without detriment to the quality of prediction.

#### Modeling the Three-way Referential Choice

The set of candidate activation factors employed in this study is derived from the vast tradition of studies on basic referential choice. We have reached a significant success in predicting the basic choice. Now, what governs the second-order choice between the types of full noun phrases, that is, proper names and descriptions? Studies of these issues are relatively few (cf. Anderson and Hastie, 1974; Arutjunova, 1977; Seleznev, 1987; Ariel, 1990; Vieira and Poesio, 1999; Enfield and Stivers, 2007; Helmbrecht, 2009; Heller et al., 2012). We have experimentally applied our set of factors to the three-way choice between third person pronouns, proper names, and descriptions. The results can be seen in **Table 8.** The baseline is the frequency of descriptions, the most frequent referential option.

**Table 8:** Prediction of the three-way referential choice.

Algorithm	Accuracy (%)
Baseline	38.1
C4.5 Decision tree algorithm	72.3
Logistic regression	73.5
Bagging	73.1
Boosting	75.7


The fairly high accuracy of prediction we have obtained for the three-way task is intriguing. Apparently, the factors responsible for the choice between proper names and descriptions substantially intersect with our basic set of factors. This issue requires further investigation.

Note that in the three-way task boosting again demonstrates the highest results, as it did in the two-way task. Even though the advantage of boosting over the other methods again is not statistically significant, the tendency of its good performance motivates our solution to employ this method in the subsequent part of this study. (However, if we used another algorithm, at least one of those included in our study, the difference would be minimal.)

### Discussion: Referential Choice Is Not Always Categorical

Even though the machine learning modeling was quite successful, the accuracy of prediction of the basic referential choice is still quite away from 100%. An important question arises: if we continue improving our annotation (e.g., by extending the set of factors) and tuning up the modeling procedure, can referential choice be ultimately predicted with the accuracy approaching 100%? In other words, is the 10% difference between the algorithm’s prediction and the original texts due to certain shortcomings of our methods or to some more fundamental causes? We propose that complete accuracy may not be attainable due to the nature of the process of referential choice.

Referential choice appears to not be a fully categorical and deterministic process. True, there are many instances in which only a pronoun or only a full noun phrase is appropriate, but there are also numerous instances in which more than one referential option can be used. This issue was explored in Kibrik (1999, p. 39), and the basic referential choice was represented as a scale comprising five potential situations:

(3) i. full NP only
ii. full NP, ^?^pronoun
iii. either full NP or pronoun
iv. pronoun, ^?^full NP
v. pronoun only.

**FIGURE 4:**
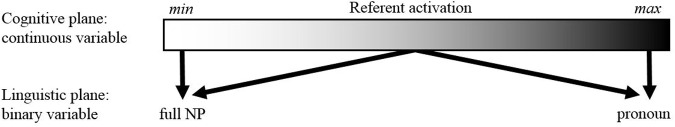
**Categorical and non-categorical referential choice**.

In (3), situations i and v are fully confident, or categorical, in the sense that language speakers would only use this particular device at the given point in discourse. Situations ii and iv suggest that, in addition to a preferred device, one can marginally use an alternative question-marked device. Finally, situation iii means free variation. In Kibrik (1999) specific referent mentions were attributed to five categories via an experimental procedure. Participants were offered modified versions of the original text, in which referential options were altered – for example, a full noun phrase was replaced by a pronoun or vice versa. Participants were asked to pinpoint infelicitous elements in the text and edit them. As a result of this procedure, some referential devices were assessed as categorical (types i, v). Other referential devices were judged partly (types ii, iv) or fully (type iii) alterable, or non-categorical. (Refer to the original publication for further details.) From the cognitive perspective, this can be interpreted as a mapping from the continuous referent activation to the binary formal distinction, as shown in **Figure 4.** That is, the formulation of the main law of referential choice, as offered in Section 1, suggests an overly categorical representation. It only captures correctly the two poles of the activation scale, but there are intermediate grades of activation in between that lead to less than categorical referential choice. The model of referential choice that we propose, as shown in **Figure 4**, differs from the well-known hierarchies of Givón (1983), Ariel (1990), and Gundel et al. (1993) in two respects. First, it explicitly recognizes a continuous cognitive variable, and second, it only focuses on the highest level distinction between full and reduced referential devices.

Non-categorical and/or probabilistic nature of referential choice has previously been addressed in a number of studies (e.g., Viethen and Dale, 2006a,b; Belz and Varges, 2007; Gundel et al., 2012; Khan et al., 2012; van Deemter et al., 2012; Engonopoulos and Koller, 2014; Ferreira et al., 2016; Hendriks, 2016; Zarrieß, 2016). For example, the well-known scale of Gundel et al. (1993) is implicational in its nature, and that is a way to partly account for the incomplete categoricity of referential choice. Krahmer and van Deemter (2012), noting that the deterministic approach dominates the field, discuss the studies by Di Fabbrizio et al. (2008) and Dale and Viethen (2010) that proposed probabilistic models accounting for individual differences between speakers. van Deemter et al. (2012, p. 18) remark that the probabilistic approach can be extended to a within-individual analysis:

Closer examination of the data of individual participants of almost any study reveals that their responses vary substantially, even within a single experimental condition. For example, we examined the data of Fukumura and van Gompel (2010), who conducted experiments that investigated the choice between a pronoun and a name for referring to a previously mentioned discourse entity. The clear majority (79%) of participants in their two main experiments behaved non-deterministically, that is, they produced more than one type of referring expression (i.e., both a pronoun and a name) in at least one of the conditions.

Overall, there is accumulating evidence suggesting that human referential choice is not fully categorical. There are certain conditions in which more than one referential option is appropriate and, in fact, each one would fare well enough. Under such conditions human language users may act differently on different occasions. If so, an efficient algorithm imitating human behavior may legitimately perform referential choice in different ways, sometimes coinciding with the original text and sometimes diverging from it. Therefore, ideal prediction of referential choice should not be possible in principle.

We have designed an experiment in which we attempt to differentiate between the two kinds of the algorithm’s divergences from the original referential choices. Of course, there may be instances due to plain error. But apart from that, there may be other instances associated with the inherently non-categorical nature of referential choice.

## Experimental Studies of Referential Variation

### Related Work

As was discussed in Section “Discussion: Referential Choice Is Not Always Categorical”, referential variation and non-categoricity is clearly gaining attention in the modern linguistic, computational, and psycholinguistic literature. Referential variation may be due to the interlocutors’ perspective taking and their efforts to coordinate cognitive processes, see e.g., Koolen et al. (2011), Heller et al. (2012), and Baumann et al. (2014). A number of corpus-based studies and psycholinguistic studies explored various factors involved in the phenomenon of overspecification, occurring regularly in natural language (e.g., Kaiser et al., 2011; Hendriks, 2014; Vogels et al., 2014; Fukumura and van Gompel, 2015). Kibrik (2011, pp. 56–60) proposed to differentiate between three kinds of speaker’s referential strategies, differing in the extent to which the speaker takes the addressee’s actual cognitive state into account: egocentric, optimal, and overprotective. There is a series of recent studies addressing other aspects of referential variation, e.g., as a function of individual differences (Nieuwland and van Berkum, 2006), depending on age (Hughes and Allen, 2013; Hendriks et al., 2014) or gender (Arnold, 2015), under high cognitive load (van Rij et al., 2011; Vogels et al., 2014) and even under the left prefrontal cortex stimulation (Arnold et al., 2014). These studies, both on production and on comprehension of referential expressions, open up a whole new field in the exploration of reference.

We discuss a more general kind of referential variation, probably associated with the intermediate level of referent activation. This kind of variation may occur in any discourse type. In order to test the non-categorical character of referential choice we previously conducted two experiments, based on the materials of our text corpus. Both of these experiments were somewhat similar to the experiment from Kibrik (1999), described in Section “Discussion: Referential Choice Is Not Always Categorical” above.

In a *comprehension experiment*, Khudyakova (2012) tested the human ability to understand texts, in which the predicted referential device diverged from the original text. Nine texts from the corpus were randomly selected, such that they contained a predicted pronoun instead of an original full NP; text length did not exceed 250 words. In addition to the nine original texts, nine modified texts were created in which the original referential device (proper name) was replaced by the one predicted by the algorithm (pronoun). Two experimental lists were formed, each containing nine texts (four texts in an original version and five in a modified one, or vice versa), so that original and modified texts alternated between the two lists.

The experiment was run online on Virtual Experiments platform^3^ with 60 participants with the expert level command of English. Each participant was asked to read all the nine texts one at a time, and answer a set of three questions after each text. Each text appeared in full on the screen, and disappeared when the participant was presented with three multiple-choice questions about referents in the text, beginning with a WH-word. Two of those were control questions, related to referents that did not create divergences. The third question was experimental: it concerned the referent in point, that is the one that was predicted by the algorithm differently from the original text. Questions were presented in a random order. Each participant thus answered 18 control questions and nine experimental questions. In the alleged instances of non-categorical referential choice, allowing both a full NP and a pronoun, experimental questions to proper names (original) and to pronouns (predicted) were expected to be answered with a comparable level of accuracy.

The accuracy of the answers to the experimental questions to proper names, as well as to the control questions, was found to be 84%. In seven out of nine texts, experimental questions to pronouns were answered with the comparable accuracy of 80%. We propose that in these seven instances we deal exactly with non-categorical referential choice, probably associated with an intermediate level of referent activation. Two remaining instances may result from the algorithms’ errors.

The processes of discourse production and comprehension are related but distinct, so we also conducted an *editing experiment* (Khudyakova et al., 2014), imitating referential choice as performed by a language speaker/writer. In the editing experiment, 47 participants with the expert level command of English were asked to read several texts from the corpus and choose all possible referential options for a referent at a certain point in discourse. Twenty seven texts from the corpus were selected for that study. The texts contained 31 critical points, in which the choice of the algorithm diverged from the one in the original text. At each critical point, as well as at two other points per text (control points), a choice was offered between a description, a proper name (where appropriate), and a pronoun. Both critical and control points did not include syntactically determined pronouns. The participants edited from 5 to 9 texts each, depending on the texts’ length. The task was to choose all appropriate options (possibly more than one). We found that in all texts at least two referential options were proposed for each point in question, both critical and control ones.

The experiments on comprehension and editing demonstrated the variability of referential choice characteristic of the corpus texts. However, a methodological problem with these experiments was associated with the fact that each predicted referential expression was treated independently, whereas in real language use each referential expression depends on the previous context and creates a context for the subsequent referential expressions in the chain. In order to create texts that are more amenable to human evaluation, in the present study we introduce a flexible prediction script.

### Human Evaluation

#### Preparation of Experimental Material: Flexible Prediction

The modeling method presented in Section “Corpus-Based Modeling” predicts referential choice at each point in discourse where a referential expression is found in the original text. For each referent, if the predicted choice at point *n* diverges from the original one, the subsequent referential expression *n+1* is again predicted by the algorithm on the basis of the original antecedent, rather than on the basis of the previous prediction. This is a traditional and valid method to generally evaluate the accuracy of the algorithm’s operation; however, in an experimental setting, where a human evaluation of the whole text is involved, such method is problematic. In order to make referential choices more natural, it is desirable to create a new version of a referential chain, such that a prediction at point *n+1* takes into account what the algorithm had predicted at point *n*.

For human evaluation, we have created a flexible modeling script. The selected referential chain is excluded from the data used for machine learning, so that the training data is kept separate from the test data. The boosting algorithm is run for each member of the chain. If there is a discrepancy between the algorithm’s choice and the original choice, it is the predicted referential expression that is used as the antecedent for the subsequent prediction. In this approach each instance of referential choice depends on all previous choices, which is more realistic from the cognitive point of view. We have made changes to the original texts according to the boosting predictions, so that new modified texts were created for each of the two evaluation studies: expert evaluation and experimental evaluation.

#### Two Stages of Human Evaluation

Human evaluation of predicted referential expressions was performed in two stages. The first stage is a rough evaluation of all the divergences of predicted referential forms from the original texts, done by a single expert. The goal of expert evaluation is to outline a distinction between crude algorithm’s errors, leading to a linguistic ill-formedness or a change in the original meaning of a text, and those divergences that may be actually acceptable for a human language user.

The second stage of human evaluation is an experiment with native speakers of English. In contrast to expert evaluation, at the stage of experimental evaluation we select a subset of divergences and present those to multiple participants.

### Expert Evaluation

#### Materials and Methods

Out of the 64 corpus texts, 48 texts demonstrated divergences from the original ones. These texts contained the total of 229 instances of divergence, including 95 predicted pronouns (instead of original full NPs) and 134 predicted full NPs (instead of original pronouns). For the purpose of expert evaluation modified versions of all 48 texts were created, with the use of the flexible script. In the modified texts, original full NPs were replaced by pronouns (with the proper number, gender, and case features), and, conversely, original pronouns were replaced by the most obvious descriptive designation of the referent (same as used in the text elsewhere), such as *the company* for ‘General Electric’ or *the president* for ‘George Bush’.

The modified texts were analyzed by one of the coauthors of this paper. As a result of text assessment, the following most common types of undoubted referential errors were detected: use of a full NP in the context of syntactic anaphora and non-cataphoric third person pronouns at the beginning of a referential chain. Example (4) demonstrates a text excerpt with two predictions not matching the referential expressions found in the original text. The original referential expressions are underlined, and the divergent predictions of the algorithm are indicated in brackets, followed by a specific referential form as used in the experiment. Prediction < 2 > was rated by the expert as potentially fitting, whereas prediction < 1 > was rated as an obvious error, namely a full NP predicted in the context of syntactic anaphora.

(4) Like Brecht, and indeed Ezra Pound, Ms. Bogart has said that < 1 > her [full NP: *the director’s*] intent in such manipulative staging of the classics is simply an attempt to make it new. Indeed, during a recent post-production audience discussion, < 2 > the director [pronoun: *she*] explained that her fondest artistic wish was to find a way to play < … >

#### Results

The analysis detected 26 undoubted referential errors that constituted 11% of all divergent predictions and just 1.2% of all referential choices predicted by the algorithm (that is, of 2248 anaphors, see **Table 3**).

Results of expert evaluation suggest that, from a reader’s point of view, replacement of an original referential expression by the predicted one mostly does not lead to an obvious referential error. In the texts analyzed, the traditionally measured accuracy of prediction was 90%; however, it appears that, out of the remaining 10%, there were only 1.2% of instances in which a predicted referential expression was rated as completely inappropriate. We interpret this finding as follows: it is not all of divergences of algorithm’s prediction from the original texts that are due to error, and the traditional approach to measuring the accuracy of prediction may conceal the difference between the natural variability of referential choice and inaccurate algorithm performance.

### Experimental Evaluation

The aim of experimental evaluation was to see how native speakers of English comprehend texts with referential choices, modified in accordance with the algorithm’s predictions. If divergent predictions are appropriate referential options, we expect no significant difference in the participants’ ability to understand the original and the modified texts, and to answer questions about the referents. If the predicted referential option is inappropriate, we expect that comprehending a modified text is harder. We measure the ease or difficulty of comprehension by the participants’ correctness in answering questions about referents, as well as by the participants rating the difficulty of each question.

#### Materials and Methods

Due to the nature of the natural texts in the corpus, we had to apply a number of restrictions on the material to make it suitable for experimental evaluation. We have selected modified texts from the corpus according to the following criteria:

1. length no less than 140 words, to avoid particularly short texts
2. length not exceeding 260 words, in order to control for the duration of the experiment
3. divergence-containing referential chains that involve at least three anaphors, in order to check the implementation of the flexible script
4. only one divergence per referential chain
5. predicted pronoun in place of an original full NP.

The two latter criteria call for explanatory comments. The decision to select referential chains with one divergence from the original was made in order to have modified and original texts differ in exactly one point, and thus to control for the number of factors involved. The application of the flexible script ensured that, in a given referential chain, after the predicted pronoun all subsequent referential choices did not diverge from the original. Note that the use of the flexible script was still useful: in the earlier comprehension experiment (Khudyakova, 2012) the difficulty of comprehending certain experimental texts could be attributed to the mismatch between the predicted divergent pronoun and the subsequent context. Using the flexible script helped to avoid such situations.

As for the last criterion, we had two reasons for only including the instances of underspecification by the algorithm. First, in the instances of overspecification the exact form of a referential expression (e.g., choice of a nominal lexeme, attributes, etc.) is not generated, and therefore a modified text would contain a referential choice supplied by a human experimenter. Second, this kind of divergence is much more informative: as was discussed in Section “Results”, class imbalance leads to the algorithms’ general predisposition to predict full NPs.

The resulting experimental set, containing all the texts matching the selection criteria, consisted of six texts. (Note that all of the obvious errors identifed at the stage of expert evaluation were filtered out due to the selection criteria.) We created a modified version of each text: the original full NP was replaced by a predicted pronoun. Then two experimental lists were created, each containing six texts, of which three were in a modified version and three texts were the original ones from the corpus.

Three questions for each text were formulated: one experimental and two control ones. Each experimental question concerned a relevant referential device, that is, one of those for which a pronoun was predicted by the algorithm. WH-words (*who, whom, whose*, or *what*) were used in the experimental questions. One of the control questions was also a WH-question, while the other one was a polar (yes-no) question.

An example of a text can be seen in (5), with the original full NP underlined, followed by the predicted pronoun in brackets. The three questions are provided below with correct responses in parentheses, and the experimental question is underlined.

(5) Milton Petrie, chairman of Petrie Stores Corp. said he has agreed to sell his 15.2% stake in Deb Shops Corp. to Petrie Stores. In a Securities and Exchange Commission filing, Mr. Petrie said that on Oct. 26 Petrie Stores agreed to purchase Mr. Petrie’s [his] 2,331,100 Deb Shops shares. The transaction will take place tomorrow. The filing said Petrie Stores of Secaucus, N.J. is purchasing Mr. Petrie’s Deb Shops stake as an investment. Although Petrie Stores has considered seeking to acquire the remaining equity of Deb Stores, it has no current intention to pursue such a possibility, the filing said. Philadelphia based Deb Shops said it saw little significance in Mr. Petrie selling his stock to Petrie Stores. We do not look at it and say, ‘Oh my God, something is going to happen,’ said Stanley Uhr, vice president and corporate counsel. Mr. Uhr said that Mr. Petrie or his company have been accumulating Deb Shops stock for several years, each time issuing a similar regulatory statement. He said no discussions currently are taking place between the two companies.

Whose shares will Petrie stores purchase? (Mr. Petrie’s)

Where are Deb Shops based? (Philadelphia)

Does Stanley Uhr work for Petrie stores? (no)

The experiment was run online using the Ibex Farm platform^4^ Each text appeared on the screen one line at a time. In the experiment we presented the texts as closely to their original appearance in the newspaper as possible, so the line length was approximately 40 characters, which matches the size of a column in Wall Street Journal. In order to see the following line of the text a participant had to press a button. Prior text did not disappear from the screen. The self-paced reading design was chosen to ensure that the participants would pay attention to all elements of the experimental texts. After the participants finished reading the text, three questions, one experimental and two control ones, appeared on the screen in a randomized order, one at a time, with the text remaining visible. Since the experimental texts are quite hard for readers (all the texts are rated as “difficult to read” or “college-level” by standard readability metrics, see **Table 9** for details), answering questions without the texts remaining available could result in an excessive rate of errors.

**Table 9:** Readability indices for the texts used in the experimental evaluation of referential choice.

Text	Flesch Reading Ease score (Kincaid et al., 1975)	Gunning Fog (Gunning, 1968)	Flesch-Kincaid Grade Level (Kincaid et al., 1975)	The Coleman-Liau Index (Coleman and Liau, 1975)	The SMOG Index (McLaughlin, 1969)	Automated Readability Index (Senter and Smith, 1967)
	30-49: Difficult	Grade level				
	50-59: Fairly difficult	(1 to 12 correspond to school grades, 13 and higher to college levels)				

1	36.0	17.5	14.1	14.0	13.7	15.7
2	58.1	12.3	9.7	10.0	9.4	9.5
3	43.5	17.2	14.1	9.0	12.9	14.1
4	38.0	18.1	15.2	11.0	13.7	15.8
5	36.9	15.6	14.0	11.0	12.7	13.7
6	46.7	13.7	11.7	10.0	12.4	11.1
Average	43.2	15.7	13.1	10.8	12.5	13.3


Participants were also asked to rate the difficulty of each question on a 5-point scale, ranging from 1 “very easy” to 5 “very hard”.

Twenty four people, including 17 females and 7 males, aged 25 to 36, took part in the experiment. All participants were native speakers of English with college-level education and explicitly stated their willingness to voluntarily participate in the experiment.

#### Results

Experiment participants answered 18 questions each, that is three questions per text. All participants provided 15 or more correct responses; the number of incorrect responses by participant is summarized in **Table 10.**

**Table 10:** Numbers of correct and incorrect responses given by participants.

Number of incorrect responses (out of 18)	Number of participants
0	6
1	6
2	9
3	3


Questions can be divided into three groups: experimental questions to original referential expressions, experimental questions to modified (predicted) referential expressions, and control questions. All questions were answered correctly by at least 75% of the participants. The numbers and percentages of correct responses are shown in **Table 11.** The ratings are shown in the right hand part of **Table 11.**

**Table 11:** Numbers of correct responses to each question in the experiment and difficulty ratings.

Question group	Question number	Correct responses		Ratings		
			
		N out of 12	% of all responses	Mean	Median	Mode
Experimental questions, original referential expression	1	11	91.67	2.83	3	3
	2	10	83.33	2.67	2.5	2
	3	11	91.67	2.83	3	4
	4	10	83.33	2.75	3	3
	5	11	91.67	2.75	3	3
	6	11	91.67	2.50	2.5	4
Experimental questions, modified referential expression	1	10	83.33	2.50	2.5	3
	2	11	91.67	2.58	3	3
	3	10	83.33	2.75	3	2
	4	11	91.67	2.92	3	3
	5	11	91.67	2.83	3	4
	6	11	91.67	2.58	3	3

		**N out of 24**	**% of all responses**	**Mean**	**Median**	**Mode**

Control questions	1 yes/no	22	91.67	2.63	2.5	2
	1 WH	23	95.83	2.67	2.5	2
	2 yes/no	22	91.67	2.83	3	3
	2 WH	23	95.83	2.92	3	3
	3 yes/no	20	83.33	2.63	3	3
	3 WH	21	87.50	2.63	3	3
	4 yes/no	21	87.50	2.67	2.5	2
	4 WH	23	95.83	2.58	3	3
	5 yes/no	18	75.00	2.67	3	3
	5 WH	22	91.67	2.67	2.5	2
	6 yes/no	21	87.50	2.67	3	3
	6 WH	22	91.67	2.67	3	3


In order to test the equivalence of correct response rates for the three groups of questions we performed the TOST (two one-sided tests) equivalence test (Schuirman, 1987) that treats the difference between groups as a null hypothesis. For the equivalence threshold set at 10%, the test yielded that the experimental groups of responses (modified vs. original referential forms) were equivalent (*p* = 0.001, CI 90% [-4.5, 4.5]). This demonstrates that, statistically, the overall perceived correctness does not differ for the original and modified texts. The same test was applied to check for statistical equivalence of correct response rates to experimental questions (about the original expressions), as opposed to responses to control questions. The two groups were proved to be statistically equivalent for the threshold of 10% (*p* = 0.001, CI 90% [-5.1, 3.7]).

We thus did not detect differences between the human understanding of original and predicted referential expressions, and it appears that in the analyzed texts instances of divergent referential choice occur in the situations in which either a full NP or a pronoun is appropriate from a human language user’s perspective.

### Discussion

The results of both evaluation studies support the idea that the divergent referential options predicted by the algorithm mostly occur in the situations in which a human language user accepts either referential form, or processes both the original and the predicted forms equally well.

Expert evaluation suggests that the majority of discrepancies between the original texts and the algorithm predictions do not result from outright algorithm errors, but rather can be interpreted as equally appropriate referential expressions. The results of the experimental evaluation suggest that, in the selected texts, replacement of a full NP by a pronoun, as predicted by the algorithm, does not lead to increased comprehension difficulty, measured both objectively (correctness of responses) and subjectively (question difficulty ratings). Though the nature of experimental evaluation does not allow us to test all the instances of divergent predictions, the observed results demonstrate that both the original and the predicted referential forms may quite often be equally appropriate.

In experimental evaluation, participants answered questions about the original and modified texts and thus played the role of discourse interpreters, rather than producers. A certain caution must be exercised when extending the experiment results to referential choice, which is a part of discourse production. One might possibly argue that, even if readers allow for more than one referential option, human writers would still perform referential choice in a categorical and deterministic way. Clearly, further experimentation is required, putting human participants in a position closer to that of a discourse producer. Note, however, that the earlier editing experiment reported in Section “Related Work” (Khudyakova et al., 2014) also indicated a strong non-categorical effect in a situation imitating human discourse production.

Overall, we propose that human evaluation of machine learning results provides more precise information about the appropriateness of referential choice prediction than the traditional accuracy measurement. Only human language users can detect whether the divergent referential choices offered by the machine are actually appropriate, and thus provide us with a clear understanding of the algorithm’s error rate.

## General Discussion

The approach we used in this study is characterized by several major conceptual elements. First, we mostly focused on the basic referential choice between full and reduced referential devices, also looking occasionally into the second order distinction between two kinds of full NPs: proper names and descriptions. Second, as is suggested by extensive prior research, we took into account a multiplicity of factors affecting referential choice. The factors we have analyzed fall into two major groups: stable referent properties and flexible factors associated with the discourse context, that latter involving several distances from an anaphor to the antecedent. Third, we used a corpus of texts, sufficient from a statistical point of view. The corpus was annotated for reference and for multiple parameters that potentially can serve as factors of referential choice. Fourth, we employed a cross-methodological approach, combining the corpus-based computational modeling and experimentation with human participants.

Two main findings result from this study, the first one concerned with computational prediction of referential choice, and the second one with the limits of such prediction. Below we summarize them in turn.

Machine learning techniques were used to predict referential choice at each point where an anaphor occured in the corpus texts. In most previous machine learning-based studies of referential choice authors primarily used decision trees. In contrast, our study is characterized by the use of a wide variety of machine learning algorithms, including classifier compositions. Trained models provided almost 90% accurate prediction of referential choices and demonstrated that machine learning algorithms can imitate referential choices made by human language users with substantial success. We also explored the cumulative and individual contribution of various factors to the resulting referential choice.

In spite of the relatively successful modeling results, prediction accuracy did not approach 100%, and this raised the question of whether complete accuracy is attainable. In order to address this question, we used experimentation with human participants. We submitted the results of modeling to human judgment and assessed the divergences between the original and predicted referential choices as appropriate or inappropriate from the language users’ point of view. Experiment results suggest that there are numerous instances in which referential options are equally appropriate for human participants. Accordingly, many of the algorithm’s failures to predict referential choice exactly as in original texts may be due not to plain error but to inherently not fully categorical nature of referential choice. Even a perfect algorithm (or, for that matter, another human language user, or even the same language user on a different occasion) could not be expected to necessarily make the choice once implemented in a text. In other words, a certain degree of variation must be built into a realistic model of referential choice. Even if the algorithm learns to imitate non-categorical referential choice (cf. examples of non-deterministic REG algorithms in van Deemter et al., 2012), mismatches between the algorithm’s prediction and the original text would be inevitable.

A few notes are in order regarding the theoretical context of this study. Following many other students of discourse reference (Chafe, 1976; Givón, 1983, and numerous later studies), we suppose that referential choice is immediately governed by a referent’s status in the speaker’s cognition. In particular, more attenuated forms of reference are used when the referent is more salient or more activated for the speaker/writer. According to the model assumed in this study, the cognitive component responsible for referential choice is activation in working memory, and different levels of referent activation are responsible for using either a reduced or a full referential device (**Figure 1**). In this model, the linguistic factors affecting referential choice are interpreted as activation factors. Operating in conjunction, they contribute to a referent’s current activation, which, in turn, determines referential choice. In some of our previous studies (Kibrik, 1996, 1999) referent’s summary activation was computed numerically and served as an explanatory component. In the present study, the activation component is not technically implemented, as standard computer modeling techniques only provide information on the mappings from linguistic factors to referential choice. Nevertheless, we believe that it is important to keep the cognitively realistic picture in mind, even if one has to remain at the level of form-to-form mappings.

The same applies to the issue of incomplete categoricity of referential choice. We demonstrated that human language users accept more than one referential option in many contexts. One can remain at the level of such observation, but it is interesting to inquire into the causes of non-categoricity. The cognitive model assumed in this study offers a plausible explanation to this phenomenon: variation of referential options occurs in the case of intermediate referent activation; see an amendment to our cognitive model in **Figure 4.** The conclusion on the not fully categorical nature of referential choice appears particularly relevant in the contemporary context of reference studies. There is a growing interest to the variation in the use of referential expressions both in computational studies and in experimental psycholinguistics (see multiple references in Sections “Discussion: Referential Choice Is Not Always Categorical” and “Related Work”), and this study contributes to the duscussion of the possible kinds and causes of such variation. The outcome of this study thus provides support to the previously expressed idea that “non-determinism should be an important property of a psychologically realistic algorithm” (van Deemter et al., 2012, p. 19).

There are several avenues for further development of the present approach in future research. As pointed out above, machine learning algorithms normally only give access to the input layer (activation factors) and the output layer (referential choice prediction), the internal working of the algorithms remaining hidden. We would like to reinstate the cognitive interpretation that is the degrees of activation that result from the activation factors in conjunction and directly map onto referential choice. One way how this can be done is associated with some algorithms’ (e.g., logistic regression) capacity to evaluate the contribution of various factors and the certainty of prediction, which can be interpreted as activation factors and summary activation level, respectively. This can also be a path to training the algorithms to model non-categorical referential choice.

The cognitive model shown in **Figure 1** is simplified in that it leaves out the filter of referential conflict, or ambiguity, that modulates referential choice after referent activation is computed (see Fedorova et al., 2010a; Kibrik, 2011; Fedorova, 2014). Sometimes a reduced referential device is filtered out because it creates a potential ambiguity for the addressee, for the reason that there is more than one highly activated referent. As of now, some of the referential conflict-related factors, such as gender and distance in all markables, are taken into account in our modeling study, but they are interspersed among the activation factors. We intend to clarify the distinction between referent activation and the referential conflict filter in future research.

In our modeling study, there is probably space for tuning up certain activation factors, which may lead to some further improvement of prediction. As was pointed out in Section “Human Evaluation”, we detected some algorithm errors, such as overspecification in the context of syntactic anaphora or underspecification at the beginning of a referential chain. These kinds of errors can be fixed by modifying the set of factors.

The set of factors responsible for the basic referential choice turned out quite efficient in predicting the second-order choice between descriptions and proper names (end of Section “Results”). A more focused search for factors directly related to this choice is in order. Also, the proposed approach can be extended to further details of referential choice, such as varieties of attributes in descriptions, as well as less frequent referential options, e.g., demonstratives. We also believe that our approach can be used in the domains of language production other than referential choice.

In this study we looked at written discourse, as a well-controlled testing ground for sharpening the methods of cognitive and computational modeling and as the material easily lending itself to various kinds of manipulation. We assume that, in spite of the special character of newspaper texts, written discourse is created on the basis of general cognitive principles of discourse production, including referential choice, and that the discovered regularities can in principle be extended to other types of language use. Nowadays, linguistic research is opening up new horizons, including interest in interactive face-to-face communication, visual context, and multimodality. All of these developments are also relevant to the study of referential choice, see e.g., Janarthanam and Lemon (2010), Viethen (2011), de Ruiter et al. (2012), and Hoetjes et al. (2015). The theoretical and methodological approach, developed here on the basis of written texts, can also be applied to a wide range of discourse types, including various genres, spoken discourse, conversation, and multimodal interaction.

## Author Contributions

AK has conceived the general design of the study, developed the theoretical framework, selected the corpus for analysis, put together the team, allocated the assignments to coauthors, formulated the general structure of the paper, wrote Sections “Introduction” and “General Discussion”, drafted “Corpus-Based Modeling”, and edited the whole text. MK worked substantially on the corpus, developed the annotation scheme, organized the work of student assistants, designed the experimental part, conducted the experiment, and wrote Section “Experimental Studies of Referential Variation”. GB provided expertise on machine learning, conducted multiple modeling studies, helped to plan the whole study, wrote parts of Sections “Corpus-Based Modeling” and “Experimental Studies of Referential Variation”. AL provided expertise on discourse annotation, natural language generation, psycholinguistic experimentation, wrote literature surveys, complied the bibliography, performed technical editing of the paper, provided big input on all aspects of the paper. DZ worked on the corpus, organized the work of student assistants, wrote “Corpus-Based Modeling”, performed technical editing of the paper, provided big input on all aspects of the paper. All coauthors participated in developing the design of the study, acquiring the data, analyzing data, writing up the manuscript, contributed to multiple manuscript revision throughout all of stages of paper preparation.

## Conflict of Interest Statement

The authors declare that the research was conducted in the absence of any commercial or financial relationships that could be construed as a potential conflict of interest.

The reviewer CL and handling Editor declared their shared affiliation, and the handling Editor states that the process nevertheless met the standards of a fair and objective review.

## Footnotes

1 No zero symbols are introduced into the corpus for the purposes of annotation. Instead, we annotate reference on a verb form of which a zero is the subject; cf. this kind of annotation on *to force* and *sprawling*, as shown in **Figure 3.**

2 http://www.cs.waikato.ac.nz/ml/weka

3 https://virtualexs.ru

4 Drummond, A. Ibex Farm. Available at: http://spellout.net/ibexfarm.
